# Accurate modulation of photoprinting under stiffness imaging feedback for engineering ECMs with high-fidelity mechanical properties

**DOI:** 10.1038/s41378-022-00394-y

**Published:** 2022-06-02

**Authors:** Xin Li, Huaping Wang, Xinyi Dong, Qing Shi, Tao Sun, Shingo Shimoda, Qiang Huang, Toshio Fukuda

**Affiliations:** 1 Intelligent Robotics Institute, School of Mechatronical Engineering, Beijing Institute of Technology, Beijing, 100081 China; 2 The Key Laboratory of Biomimetic Robots and Systems (Beijing Institute of Technology), Ministry of Education, Beijing, 100081 China; 3 School of Medical Technology, Beijing Institute of Technology, Beijing, 100081 China; 4 Intelligent Behavior Control Collaboration Unit, RIKEN Center of Brain Science, 463-0003 Nagoya, Japan

**Keywords:** Microengraving, Electrical and electronic engineering

## Abstract

Engineered extracellular matrices (ECMs) that replicate complex in-vivo features have shown great potential in tissue engineering. Biocompatible hydrogel microstructures have been widely used to replace these native ECMs for physiologically relevant research. However, accurate reproduction of the 3D hierarchical and nonuniform mechanical stiffness inside one integrated microstructure to mimic the complex mechanical properties of native ECMs presents a major challenge. Here, by using digital holographic microscopy (DHM)-based stiffness imaging feedback, we propose a novel closed-loop control algorithm to achieve high-accuracy control of mechanical properties for hydrogel microstructures that recapitulate the physiological properties of native ECMs with high fidelity. During photoprinting, the photocuring area of the hydrogel is divided into microscale grid areas to locally control the photocuring process. With the assistance of a motorized microfluidic channel, the curing thickness is controlled with layer-by-layer stacking. The DHM-based stiffness imaging feedback allows accurate adjustment of the photocuring degree in every grid area to change the crosslinking network density of the hydrogel, thus enabling large-span and high-resolution modulation of mechanical properties. Finally, the gelatin methacrylate was used as a typical biomaterial to construct the high-fidelity biomimetic ECMs. The Young’s modulus could be flexibly modulated in the 10 kPa to 50 kPa range. Additionally, the modulus gradient was accurately controlled to within 2.9 kPa. By engineering ECM with locally different mechanical properties, cell spreading along the stiff areas was observed successfully. We believe that this method can regenerate complex biomimetic ECMs that closely recapitulate in-vivo mechanical properties for further applications in tissue engineering and biomedical research.

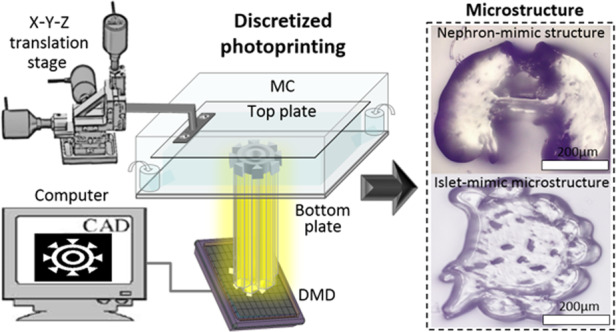

## Introduction

The extracellular matrix (ECM), as an essential part of biological tissues, provides an important mechanical support environment for the life behaviors of cell groups. Typically, the mechanical stiffness of different human tissues varies over a large range. For example, the Young’s modulus of prostate tissue is approximately 90 kPa, while breast tissue has a Young’s modulus of only 8 kPa^1^. This different mechanical stiffness among various tissues induces different physiological expressions of cell populations^2^, such as secretion and interaction, and thus exhibits different biological functions on the macroscopic scale^3^. Additionally, the high-resolution stiffness changes among local microscale areas within a single tissue also cause the behavioral differences at the cell level. Lo et al. demonstrated that the 3T3 cells can migrate from soft to stiff areas^4^. Marg et al. observed that neurons branch more on the softer substrates^5^. Therefore, when engineering the ECM, there is an urgent need to be able to modulate the mechanical stiffness over a large span while accurately controlling the stiffness gradient.

Biocompatible polymers such as UV-curable hydrogels are frequently used to engineer the biomimetic ECM in vitro^6^. Consequently, a variety of three-dimensional (3D) bioprinting techniques, such as 3D laser lithography^7^, photomold patterning^8^, and digital light processing (DLP)^9^, have emerged. These bioprinting techniques mainly focus on the optimization of printing shape, which has achieved breakthrough progress in accurately reproducing the 3D morphology of native tissues. However, due to a lack of the ability to effectively detect the mechanical stiffness, it is still a tremendous challenge to dynamically modulate the mechanical properties of biomimetic ECM at the microscale. Atomic force microscopy (AFM) as a mechanical measurement tool can flexibly determine the mechanical properties of cells and other biological materials. Cha et al. performed the stiffness measurements on gelatin methacrylate (GelMA) microgels by using AFM-assisted nano-indentation^10^. Liu et al. employed AFM to characterize the Young’s modulus of the fabricated pol(ethylene glycol) diacrylate (PEGDA) microstructures^11^. The measuring accuracy of the AFM depends on the size of the nanoindentation. However, as a kind of complicated equipment, it is difficult to combine the 3D bioprinting system with AFM so that an adjustable Young’s modulus can be obtained in real-time as feedback during the ECM fabrication to regulate the mechanical properties at the microscale. Suitable fabrication techniques to enable real-time tunable control of mechanical properties for high-fidelity fabrication of biomimetic ECM are thus still needed.

In fact, the stiffness of polymers correlates linearly with the density of the structure at the microscale^12^. If the polymer density in every microscale area can be detected in real-time during fabrication, the dynamic changes in stiffness can be obtained. As a typical polymer, the UV-curable hydrogel’s structural density can be characterized by the crosslinking network density, which is determined by the photocuring degree^13^. Askadskii et al. found that the local refractive index of hydrogels can reflect their own crosslinking network density^14^, so that there is a mapping relationship between the local stiffness and refractive index. Thus, by observing the change in the refractive index of the structure, indirect visualization and sampling of the real-time stiffness can be achieved. To measure the refractive index of a structure, techniques including precision goniometry (PG)^15^, abbe refractometry (AR)^16^, and digital holographic microscopy (DHM)^17^ are commonly utilized. Among these techniques, DHM provides an optimal solution to measure the refractive index, with the advantage of real-time, in-situ, and flexible detection. It is promising to employ DHM monitoring for the modulation of mechanical properties during 3D printing.

In this paper, we propose a novel closed-loop control algorithm with stiffness imaging feedback for the dynamic modulation of hydrogel mechanical properties during the photoprinting of high-fidelity biomimetic ECM. According to the shape of the customized ECM, the fabrication area is divided into microscale grid areas by a projection microstereolithography (PμSL) system for the local photocuring process. With a motorized microfluidic channel, the photocuring thickness of the microstructure is precisely controlled in a layer-by-layer stacking manner. The DHM is integrated into the PμSL system to observe the refractive index of the structure and consequently sample the Young’s modulus of the microstructure during the photocuring process. With DHM-based stiffness imaging feedback, the mechanical properties of every grid area are accurately modulated to achieve a large span and high resolution by controlling the photocuring degree of the hydrogel. Thus, the high-fidelity biomimetic ECM is obtained with a hierarchical 3D architecture and high-accuracy mechanical properties. With this method, we anticipate that 3D bioprinting will be able to reproduce high-precision morphologies and high-fidelity mechanical properties consistent with native tissue, from part to whole, for special artificial tissue.

## Results and discussion

### Experimental setup

**Fig. 1:**
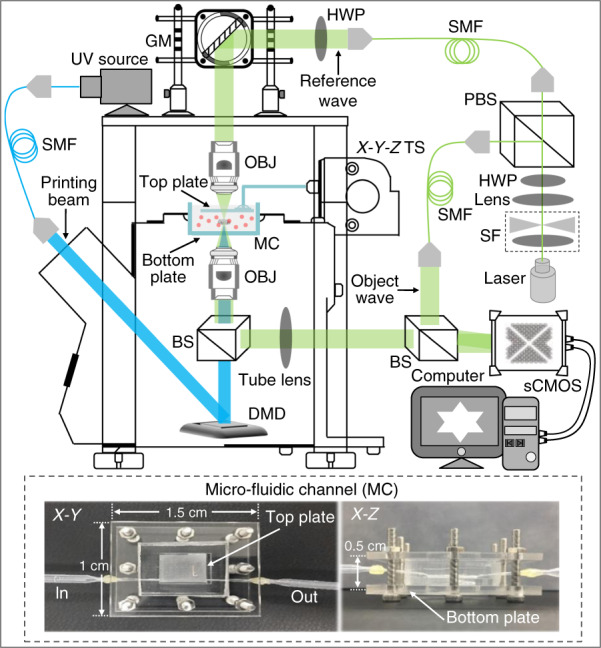
Stiffness imaging feedback-based PμSL system. TS Translation stage, BS Beam splitter, PBS Polarizing beam splitter, DMD Digital micromirror device, SMF: Single mode fiber

The experimental setup by which biomimetic ECMs with high-accuracy nonuniform mechanical properties were produced under software control for the study of cell behaviors is shown in Fig. 1. In the PμSL system, a digital micromirror device (DMD, ViALUX, Germany) with a matrix of 1024×768 individually addressable micromirrors projects a light pattern onto UV-curable hydrogel for the photocuring process of biomimetic ECM (ref. 18). Therefore, by independently controlling the projection duration of each micromirror, the photocuring degree of the local areas of the bioprinting material can be controlled, and therefore the mechanical properties of the biomimetic ECM can be locally modulated. Additionally, the motorized X-Y-Z translation stage-assisted microfluidic channel plays an important role during the fabrication of biomimetic ECM (Fig. 1). The top plate connected with the translation stage can be accurately moved and positioned along the three directions of X-Y-Z in the microfluidic channel by controlling the motors. When the microfluidic channel is filled with a UV-curable hydrogel, the top plate is positioned in the Z direction using the motors to limit the distance between the top and bottom plates, so as to accurately control the printing thickness of the biomimetic ECM.

To measure the mechanical properties of biomimetic ECM during fabrication, we built a DHM-based stiffness imaging system. A schematic of the hardware setup is shown in Fig. 1^19,20^. A 532-nm single longitudinal-mode laser (MSL-FN-532-150 mW, Changchun New Industries Optoelectronics Technology) is used as the illumination source for the stiffness imaging. The 532-nm light is then filtered by the spatial filter to obtain the plane light. A polarization-dependent beam splitter (CCM1-PBS251, Thorlabs) divides the plane light into two beams, of which one beam illuminates the sample on the microfluidic channel, while the other serves as a reference. The illuminating beam is controlled by galvo mirrors (1×GVS211/M, Thorlabs) to pass through the sample and the detection objective (4×/0.13, ApoN, Olympus) at a perpendicular angle and finally combined with the reference beam by the beam-splitter (BS) to generate a hologram on an sCMOS camera (ORCA-Flash 4.0 V3, Hamamatsu), providing sufficient total photon flux within 50 μs of the exposure time. Numerical reconstruction of the hologram provides the local mechanical properties of the sample as real-time feedback to the PμSL system.

In this study, GelMA was used as the experimental material to demonstrate the highly controllable fabrication of a biomimetic ECM with high-fidelity mechanical properties. GelMA is a UV-curable hydrogel with excellent biocompatibility and is commonly used to fabricate biomimetic microstructures in many bioengineering applications^21^. To obtain the GelMA prepolymer solution, lithium pheny1-2,4,6-trimethyl-benzoyl-phosphinate (LAP, Allevi, USA) and frozen GelMA were dissolved in Dulbecco’s modified Eagle’s medium (DMEM) at 37 °C. By stirring, the final prepolymer solution containing 20% (w/v) GelMA and 1% (w/v) LAP was formed^22^.

### DMD-based stiffness imaging

**Fig. 2:**
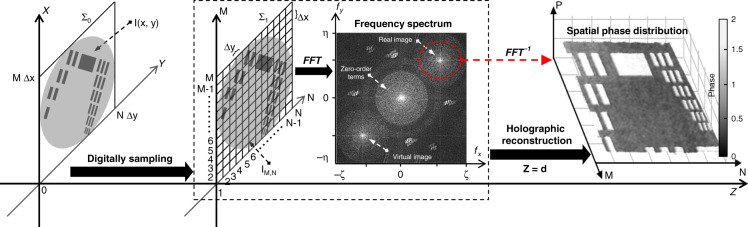
Grid division of hologram and angular spectrum method-based holographic reconstruction

The PμSL system integrated with stiffness imaging feedback is a significant improvement compared with the traditional methods used to photoprint biomimetic ECM. The high-accuracy mechanical properties in local microscale areas can be produced by accurately adjusting the photocuring degree of every local area in real time under closed-loop control, which enables the highly controllable fabrication of biomimetic ECM with high fidelity. For the UV-curable hydrogel microstructure, the structural stiffness is related to its refractive index. Therefore, to characterize the mechanical properties of the microstructure, the refractive index of the structure should be primarily measured. The DHM allows us to probe the refractive index of the microstructure in real time during fabrication. In holography, the recorded intensity *G(x,y)* of the hologram is the square module of the amplitude superposition of the object and reference waves. It is given by^23^

1 $$\begin{array}{*{20}{c}}{G(x,y) = \left| {O\left( {x,y} \right)} \right|^2 \,+\, \left| {R\left( {x,y} \right)} \right|^2 \,+\, O\left( {x,y} \right)R^ \ast \left( {x,y} \right)}\\ { + O^ \ast \left( {x,y} \right)R\left( {x,y} \right)}\end{array}$$

where *O*(*x,y*) and *R*(*x,y*) are the intensities of the optical field of the object wave and reference wave, respectively. * signifies the complex conjugate. The first two zero-order terms represent the intensities of the reference and object waves and, therefore, they do not provide the spatial information about the object optical field. The penultimate and last terms provide the spatial frequency of the recorded hologram and are responsible for the virtual and real images, respectively. Because the detection plane of the sCMOS camera is composited with a discrete pixel array, the continuously distributed intensity *G(x,y)* in the hologram is digitally sampled by the sCMOS camera to form the discrete grid areas, as shown in Fig. 2. Thus, the hologram can be represented by an *M* by *N* matrix *G*_*M,N*_, where each element is,

2 $$G_{M,N}\left( {m,n} \right) = G\left[ {m\Delta x,n\Delta y} \right]$$

where *m* = 1, 2,…, M; and *n* = 1, 2,…, N; and Δ*x* and Δ*y* are the pixel sizes of the sCMOS camera.

The holographic reconstruction can be implemented by numerically propagating the optical field along the *Z* direction using the angular spectrum method, which has a significant advantage in that a minimum reconstruction distance is not required^24^ (Fig. 2). Because the third term on the right side of Eq. (1) contains the object information, the digitally holographic representation of the sample can be described as:

3 $$G_{M,N} = {{{\mathrm{O}}}}_{M,N} \times {{{\mathrm{R}}}}^ \ast _{M,N}$$

where *O*_*M,N*_ and *R*_*M,N*_ are the matrix forms of *O(x,y)* and *R(x,y)*, respectively. Therefore, the angular spectrum *A*_*M,N*_*(ζ,η;d)* at plane z = d is calculated by taking the Fourier transform:

4 $$\left\{ {\begin{array}{*{20}{c}}{A_{M,N}(\zeta ,\eta ;d) = A_{M,N}(\zeta ,\eta ;0)}\\ { \times \exp (j\frac{{2\pi d}}{\lambda }\sqrt {1 - (\lambda \zeta )^2 - (\lambda \eta )^2} )}\\ {A_{M,N}(\zeta ,\eta ;0) = F\left\{ {G_{M,N}} \right\}}\end{array}} \right.$$

where *F*{} denotes the Fourier transform; *ζ* and *η* are the spatial frequencies of the *M* and *N* directions, respectively; *λ* is the wavelength; and *d* is the propagation distance of the object wave.

Then, the reconstructed complex-amplitude distribution at plane z = d is found by taking the inverse Fourier transform as

5 $$U^d_{M,N} = F^{ - 1}\left\{ {A_{M,N}\left( {\zeta ,\eta ;d} \right)} \right\}$$

where *F*^−1^{} denotes the inverse Fourier transform. The phase distribution *φ*_*M,N*_ of the sample can be obtained from a single digital hologram by calculating the argument of the reconstructed complex-amplitude wavefront^25^:

6 $$\varphi _{M,N} = \arctan \left[ {\frac{{{\Im} \left( {U^d_{M,N}} \right)}}{{{\Re} \left( {U^d_{M,N}} \right)}}} \right]$$

where Im() and Re() denote the real and imaginary parts, respectively. Therefore, the refractive index of the sample can be described by^26^

7 $$L_{M,N} = \frac{{\lambda \varphi _{M,N}}}{{2\pi H_{M,N}}}$$

where *H*_*M,N*_ is the thickness of the sample.

**Fig. 3:**
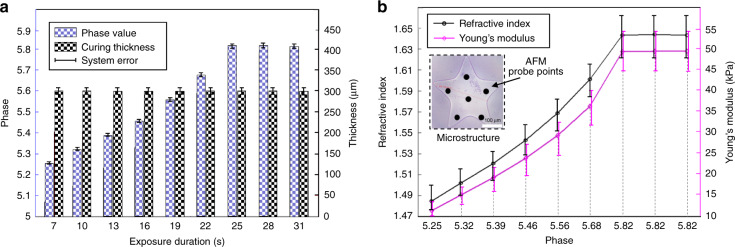
**Curve fitting of Young’s modulus**. **a** Phase distributions of microstructures under different exposure durations. **b** Measurements of Young’s modulus

To achieve high controllability of mechanical properties using the proposed fabrication system, we need to establish an equivalent model between Young’s modulus and the refractive index. Here, we used GelMA hydrogel as an example to fabricate multiple sets of pentagon-shaped microstructures with a thickness of 300 μm for fitting the equivalent model (Supplementary Fig. S1). Since the UV-curable hydrogel has a threshold dose to start the curing, the photocuring process includes three states: the noncuring state, curing state, and curing saturation. Considering that the UV exposure dose is less than 96.32 mJ/cm^2^, the/GelMA hydrogel is in the noncuring state. By increasing the exposure dose, the hydrogel transitions into the curing state. As shown in Fig. 3a, nine sets of microstructures are obtained after exposure to 13.75 mW/cm^2^ UV light for 7, 10, 13, 16, 19, 22, 25, 28 and 31 s. With Eq. (6), the phase distribution of each set of microstructures is measured by the DHM. The measured results showed that the phases of microstructures gradually increased with increasing exposure dose and did not change after the exposure dose reached 343.8 mJ/cm^2^ (the exposure dose *D* was 13.75 × 25 = 343.8 mJ/cm^2^). Thus, the phase level of the microstructure depends on the photocuring degree of the hydrogel during the curing state. The curing saturation of the GelMA hydrogel occurred after an exposure duration of > 25 s. In our system, the lateral resolution of the sCMOS camera was 6.5 μm. The minimum fabrication size was determined by the micromirror size of the DMD, which was 13.6 μm.

AFM (Bruker AFM, Camarillo, CA, USA) was then used to measure Young’s modulus of these fabricated pentagon-shaped GelMA microstructures, and the results were used to fit the equivalent model. To more accurately represent the mechanical properties of the microstructure, we took measurements at six different positions of each microstructure to obtain the mean value of Young’s modulus. The Hertz model was used to calculate Young’s modulus^27^:

8 $$\left\{ {\begin{array}{*{20}{c}} {F_{sphere} = \frac{{4ER^{1{{{\mathrm{/}}}}2}c^{3{{{\mathrm{/}}}}2}}}{{3(1 - v^2)}}} \\ {F_{cone} = \frac{{2Ec^2\tan \theta }}{{\pi (1 - v^2)}}} \end{array}} \right.$$

where *θ* is the half-opening angle of the probe tip, *F* is the acting force, *v* is the Poisson’s ratio of GelMA and equals 0.5, *E* is the Young’s modulus, *R* is the curvature radius and is equal to 10 nm, and *c* is the indentation depth. As shown in Fig. 3b, the black points represent the AFM probe positions, where the Young’s modulus of nine sets of fabricated microstructures was measured (red curve). The refractive indices of the microstructures were calculated by substituting the phase values shown in Fig. 3a into Eq. (7). Because the refractive indices were determined by the phase levels of the microstructure, the refractive indices can be adjusted by changing the photocuring degree of the hydrogel.

As shown in Fig. 3b, the mechanical properties of the GelMA hydrogel were unchanged when the hydrogel was in the curing saturation state. Therefore, we established an equivalent model during the curing state to characterize the mechanical properties of the hydrogel. The data shown in Fig. 3b were imported into a MATLAB package to find the mapping relationship between the refractive index and Young’s modulus. Table 1 lists the R-squared values under these fitting methods with exponential fitting, linear fitting, logarithmic fitting, power function fitting, and second-order polynomial fitting. Therefore, the equivalent model between the refractive index and Young’s modulus was obtained by using the linear fitting method, which can be described as:

9 $$E_{M,N} = 234L_{M,N} - 335$$

where *E(M,N)* is an *M* by *N* matrix that represents Young’s modulus of each discrete microscale grid within one hydrogel microstructure, and the unit is kPa. Thus, with Eq. (9), DHM-based stiffness imaging was indirectly achieved. In our experiment, by adjusting the photocuring degree of the GelMA hydrogel under stiffness feedback to change the refractive index, the mechanical properties of the biomimetic ECM were regionally controlled during fabrication. In fact, except for the exposure dose, the different hydrogel concentrations are also key factors that affect the stiffness of the crosslinked structure. In our method, we developed a single mapping model between the refractive index and stiffness to modulate the mechanical stiffness of hydrogels in real time. Since the refractive index is an inherent property of the crosslinked structure, it is unrelated to the concentration of the hydrogel prepared before crosslinking. Thus, this mapping model can be flexibly applied to different crosslinking process control scenarios.

**Table 1:** Fitting methods used in our experiments

Fitting methods	Exponential fitting	Linear fitting	Logarithmic fitting	Power function fitting	Second-order polynomial fitting
R-squared values	0.9846	0.9882	0.9851	0.9877	0.9879

### Control range of mechanical properties

**Fig. 4:**
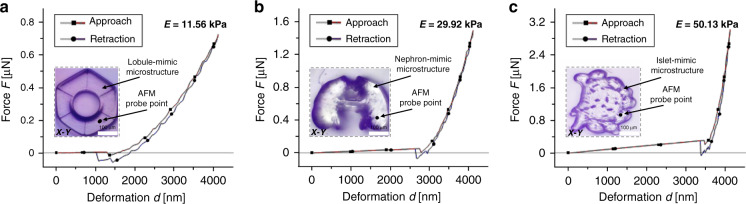
**Fabrication of biomimetic ECM**. **a** Lobule-mimic microstructure. **b** Nephron-mimic microstructure. **c** Islet-mimic microstructure

Because the mechanical properties of native tissues have a large span, our printing system is required to control Young’s modulus of the printed hydrogel over a large range, and therefore, the fabricated biomimetic ECMs can meet actual application requirements. The lobules of the liver, nephrons of the kidney, and islets of the pancreas are major tissues in the human body, and their Young’s modulus values are in the range of 0.5–16 kPa, 25–60 kPa, and 50–72 kPa, respectively. To mimic these native tissues in vitro, we designed and fabricated lobule-like, nephron-like, and islet-like microstructures using the proposed control method. Here, each type of microstructure was replicated 10 times for characterization of the mechanical properties. As shown in Fig. 4a, the lobule-mimic ECM microstructure with 15-layer cured GelMA was fabricated. The exposure duration and thickness of each layer were 7 s and 20 μm, respectively. The Young’s modulus was measured using AFM and had a mean value of 11.56 kPa. Additionally, a nephron-mimicking ECM microstructure with 15 layers of cured GelMA was fabricated. The exposure duration and thickness of each layer were 19 s and 20 μm, respectively. The islet-mimic ECM microstructure with 15-layer cured GelMA was fabricated. The exposure duration and thickness of each layer were 25 s and 20 μm, respectively. From AFM measurements, Young’s modulus of the nephron-mimic microstructure was 29.92 kPa (Fig. 4b) and that of the islet-mimic microstructure was 50.13 kPa (Fig. 4c). The Young’s modulus of these microstructures spanned a range of 40 kPa, thus demonstrating that the proposed printing method can control the mechanical properties of the microstructure over a large span.

### Accuracy analysis of mechanical properties

**Fig. 5:**
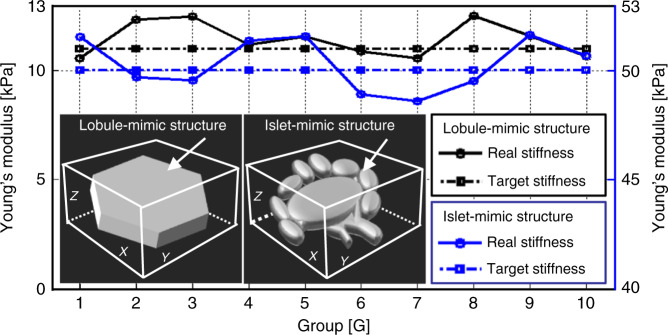
Control accuracy analyzation of the system in mechanical stiffness

On the cell level, slight stiffness changes in the local microscale areas within a single tissue can cause distinct differences in cell behavior. Thus, our printing system is required to have high printing accuracy, that is, the gradient of mechanical stiffness should be controlled to within a small range. Here, under DHM-based stiffness imaging closed-loop feedback, we replicated 10 groups of lobule-mimic ECM microstructures with a target Young’s modulus of 11 kPa. The real mechanical stiffness of each microstructure was measured using AFM, and all the values were fitted, as shown in Fig. 5. Notably, the measured values fluctuated within a range of 1.6 kPa. Similarly, in the same way, we fabricated 10 groups of islet-mimic ECM microstructures with a target Young’s modulus of 50 kPa and measured Young’s modulus of each microstructure (Fig. 5). The fluctuation range of the measured values was 2.9 kPa. Therefore, the modified PμSL system combined with real-time closed-loop feedback can significantly control the printing accuracy of the microstructure for the fabrication of high-fidelity biomimetic ECMs

### Fabrication of microstructures with complex mechanical properties

**Fig. 6:**
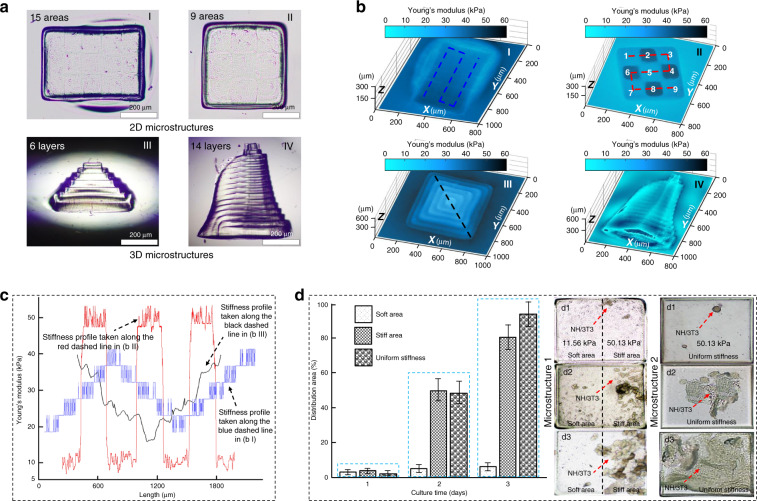
**High-accuracy control of mechanical stiffness**. **a** Microscopy images of 2D and 3D microstructures. **b** DHM-based stiffness imaging reconstruction. **c** Stiffness profiles taken along the dashed lines in (**bI**), (**bII**) and (**bIII**). **d** Quantification and distribution of NH/3T3 cells in the microstructures

Actually, the distribution of mechanical properties in native tissues is diverse and complex, which not only linearly changes in the horizontal direction but also shows hierarchical nonuniform features along the Z-direction. Here, a few representative microstructures were fabricated, and the mechanical stiffness was coded to verify the applicability of the proposed printing method. As shown in Fig. 6a, we fabricated a 2D microstructure with a linear distribution of mechanical properties. The stiffness image was reconstructed using DHM (Fig. 6b). Young’s modulus taken along the blue dashed line is fitted in Fig. 6c. The mechanical properties of the microstructure were coded into a triangular wave distribution with an error range of 4.7 kPa, thus demonstrating that the printing system can highly and accurately control the gradient of mechanical properties. In the same way, we fabricated a 2D microstructure with a square-wave distribution of mechanical properties. The reconstructed stiffness image is shown in Fig. 6b. Similarly, the Young’s modulus taken along the red dashed line was fitted in Fig. 6c. The fluctuation range was in the range of 40 kPa, which demonstrates that the printing system can modulate the mechanical properties over a large span. Additionally, we fabricated multilayer 3D microstructures using layer-by-layer stacking. The 6-layer microstructure was obtained by photocuring each layer for 10, 14, 17, 20, 22, and 23 s, respectively. The 14-layer microstructure was obtained by photocuring each layer for 24, 22, 20.5, 19, 17.5, 16, 14.5, 13, 12, 11, 10, 9, 8, and 7 s, respectively. Figure 6b shows the reconstructed 3D stiffness images of these microstructures. The mechanical stiffness gradually increased from the top layer to the bottom layer. The Young’s modulus taken along the black dashed line is fitted in Fig. 6c. Here, the fluctuation range of the modulus in the Fig. 6c is greater than 2.9 kPa. Actually, it is not only determined by the fabrication error range. In this experiment, the structure shown in Fig. 6b (II) was formed with 9 individual blocks that were tightly connected. Here, each block was fabricated in a separate photocuring cycle, which means the blocks were photocured one by one. The corresponding light scattering caused slight crosslinking reactions of the surrounding hydrogel. For example, when block 2 in Fig. 6b (II) was photocuring, the light scattering induced localized slight curing of block 1 that had already been fabricated. Since the photocuring cycle for block 1 had ended, the feedback loop could not modulate the stiffness of block 1 again. Therefore, the fluctuation range of modulus in the Fig. 6c was as high as 4.7 kP. To avoid this kind of situation, all of these blocks can be synchronously fabricated within one photocuring cycle, enabling the feedback loop to modulate every block from beginning to end, which controls the accuracy in the range of 2.9 kPa. Therefore, our printing system can achieve the large-span and high-accuracy modulation of mechanical properties during the fabrication of microstructures that closely mimic native ECMs. To investigate the influences of mechanical properties on cell behaviors, we fabricated GelMA microstructures, as shown in Fig. 6d. The NH/3T3 cells were cultured by seeding on the stiffness junction of the microstructure 1 with different mechanical properties. Here, the Young’s modulus of the soft area was 11.56 kPa, while that of the stiff area was 50.13 kPa. The image analysis software ImageJ was used to quantify the distribution of NH/3T3 cells. According to the characteristics of the adherent cells, the cells tend to settle down and grow in the stiff substrate. When the substrate is composed of soft and stiff areas, the seeded cells are more likely to migrate in the stiff areas. Therefore, the cells could spread and proliferate to cover the stiff area of microstructure 1 after 3 days of culture, but hardly proliferated in the soft area (Fig. 6d). Additionally, NH/3T3 cells were initially seeded on the surface of microstructure 2 without any variation in the mechanical stiffness. Young’s modulus of microstructure 2 was 50.13 kPa. The cells could spread and proliferate to cover the whole area of microstructure 2 after 3 days of culture. A more detailed experiment is presented in the Supplementary Fig. S2 to describe the cell behaviors. This trend is consistent with previous report that cells are more likely to spread and proliferate in the stiff areas^4^, which verified that the mechanical properties of the microstructure can affect the cell behaviors.

## Conclusion

In this study, we developed a novel closed-loop control algorithm to construct biomimetic ECMs that recapitulate complex in vivo features. DHM-based stiffness imaging was added as feedback to the PμSL system to enable highly efficient photoprinting of biomimetic ECMs with high-fidelity mechanical properties. In this system, Young’s modulus is sampled in real time using DHM-based stiffness imaging to dynamically adjust the photocuring degree of the hydrogel, and thus, the mechanical properties of biomimetic ECMs can be modulated with high accuracy. GelMA, a typical biocompatible hydrogel, was used to construct the biomimetic ECMs, and several hierarchical microstructures with locally different mechanical properties were fabricated. Young’s modulus of the microstructures could be flexibly modulated in the range of 10 kPa to 50 kPa. Additionally, the modulus gradient could be controlled to within 2.9 kPa. Therefore, the proposed photoprinting approach was able to modulate the mechanical stiffness over a large span while accurately controlling the stiffness gradient. To mimic native ECMs and to study the cell behaviors, NH/3T3 cells were seeded on the surface of the microstructures with locally different mechanical properties for culture. Cell spreading along the stiff areas was successfully observed, which verified that the mechanical properties of the microstructure affect cellular behavior. Overall, the experimental results verified that the developed closed-loop control algorithm can efficiently achieve large-span and high-resolution mechanical properties inside a single integrated microstructure to closely mimic native tissue, which has vast potential for future biomedical research.

## Materials and methods

### Photoprinting workflow with real-time feedback

**Fig. 7:**
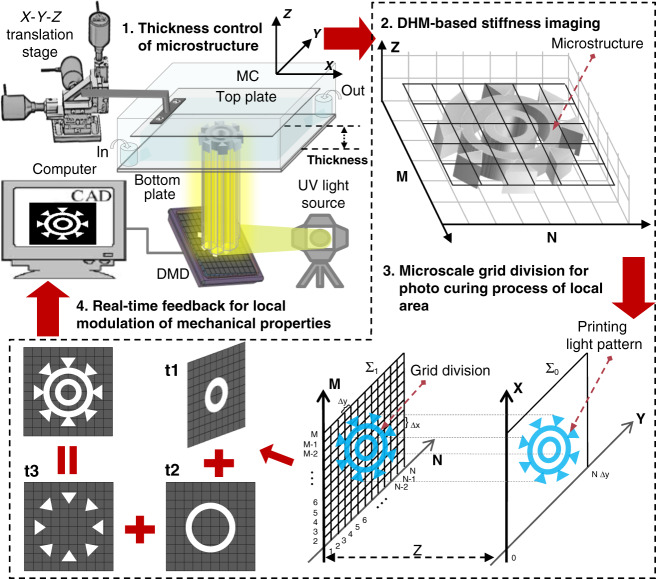
Workflow of the proposed printing strategy

By taking advantage of the equivalent model of Eq. 9, a novel printing approach was developed in which real-time feedback of stiffness imaging was used to highly accurately modulate the local mechanical properties of biomimetic ECM. We used layer-by-layer stacking to fabricate the biomimetic ECM (ref. ^28,29^). The final ECM microstructure is composed of multiple stacked layers of the cured GelMA hydrogel, where each cured layer was obtained during an independent printing cycle. As shown in Fig. 7, first, the top plate is accurately moved using a motorized X-Y-Z translation stage in the Z direction to control the distance between the top and bottom plates, which determines the printing thickness of the first printing layer. When the photocuring process is finished, the top plate lifts to a height equal to the cured layer thickness to fabricate the subprinting layer in the next printing cycle. Finally, by repeating the printing cycle, more subprinting layers are obtained and the expected biomimetic ECM is produced. The photocuring process consists of four steps in each printing cycle. In the first step, the UV light pattern is projected onto the microfluidic channel using the microlens array in the DMD chip to excite the hydrogel from the noncuring state to the curing state. In the second step, through DHM-based stiffness imaging, the printing layer is divided into microscale grid areas, and Young’s modulus of every grid area is sampled in real time by the DHM during fabrication. In the third step, the printing light pattern is also divided into microscale grids consistent with the printing layer by the DMD for individual photocuring of the local area. In the fourth step, with real-time feedback of Young’s modulus in each grid area, the photocuring degree of the local hydrogel is changed to highly accuratate modulate the mechanical Because the real-time phase value *φ*_*M,N*_ detected by the DHM is the phase sum of all layers, including the cured layers that have been fabricated and the printing layer that is being fabricated, the phase value of each cured layer should be extracted to obtain the real-time mechanical properties in the printing layer. First, we use the method of iterative subtraction to extract the phases of all layers^30^:

10 $$\left\{ \begin{array}{l}\varphi _{M,N}^0 = 0\\ \varphi _{M,N}^j = \varphi _{M,N} - \mathop {\sum}\limits_{i = 0}^{j - 1} {\varphi _{M,N}^i} \end{array} \right.$$

where *φ*^*j*^_*M,N*_ represents the phase of the printing layer in the *j*th printing cycle and *φ*^*i*^_*M,N*_ represents the phase of the cured layer in the *i*th printing cycle. With Eqs. (7) and (9), the real-time mechanical properties of the printing layer can be described as

11 $$\left\{ \begin{array}{l}L_{M,N}^j = \frac{{\lambda \times \varphi _{M,N}^j}}{{2\pi \times H_{M,N}^j}}\\ E_{M,N}^j = 234L_{M,N}^j - 335\end{array} \right.$$

where *L*^*j*^_*M,N*_ and *E*^*j*^_*M,N*_ are respectively the refractive index and Young’s modulus of the printing layer in the *j*th printing cycle.

**Fig. 8:**
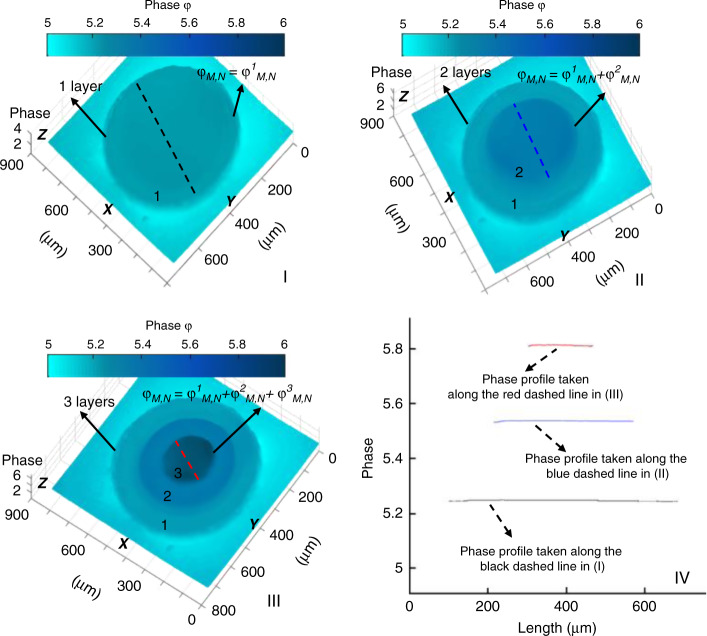
Measurement method of iterative subtraction

Moreover, *H*^*j*^_*M,N*_ represents the thickness of the printing layer in the *j*th printing cycle. Figure 8 shows the measurement method of iterative subtraction. A one-layer structure was obtained after the first printing cycle, where *φ*_*M,N*_ was equal to *φ*^*1*^_*M,N*_, as shown in Fig. 8(I). A two-layer structure was obtained after the second printing cycle, where *φ*_*M,N*_ was equal to *φ*^*1*^_*M,N*_ + *φ*^*2*^_*M,N*_, as shown in Fig. 8(II). A three-layer structure was obtained after the third printing cycle, where *φ*_*M,N*_ was equal to *φ*^*1*^_*M,N*_ + *φ*^*2*^_*M,N*_ + *φ*^*3*^_*M,N*_, as shown in Fig. 8(III). The phase taken along the dashed lines is fitted in Fig. 8(IV). Therefore, with Eq. (11), we can obtain the phase values of every layer.

## Supplementary information

Supplementary file
