# Supplementary material for: Accurate modulation of photoprinting under stiffness imaging feedback for engineering ECMs with high-fidelity mechanical properties
Source: Microsyst Nanoeng. 2022 Jun 2;8:60. doi: 10.1038/s41378-022-00394-y (PMC9163149; doi:10.1038/s41378-022-00394-y)
Supplement: Supplementary file 1 — Supplementary file [file 41378_2022_394_MOESM1_ESM.docx]

Accurate modulation of photo-printing under stiffness imaging feedback for engineering ECM with high-fidelity mechanical properties

Xin Li^1^, Huaping Wang*^1^, Xinyi Dong^1^, Qing Shi^1^, Tao Sun^1^, Shingo Shimoda^2^, Qiang Huang^1^, and Toshio Fukuda^1^

Supplementary Information

Affiliations

1. Intelligent Robotics Institute, School of Mechatronical Engineering, Beijing Institute of Technology, Beijing 100081, China.
2. Intelligent Behavior Control Collaboration Unit, RIKEN Center of Brain Science, 463-0003, Nagoya, Japan.

* Corresponding author: Huaping Wang (Email: wanghuaping@bit.edu.cn, Telephone: 86-10-68917765)


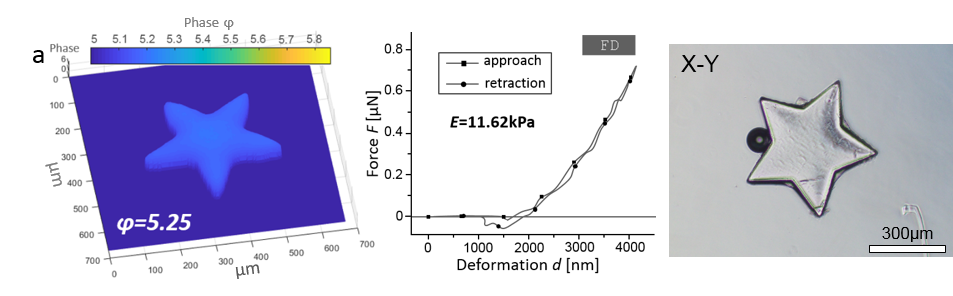


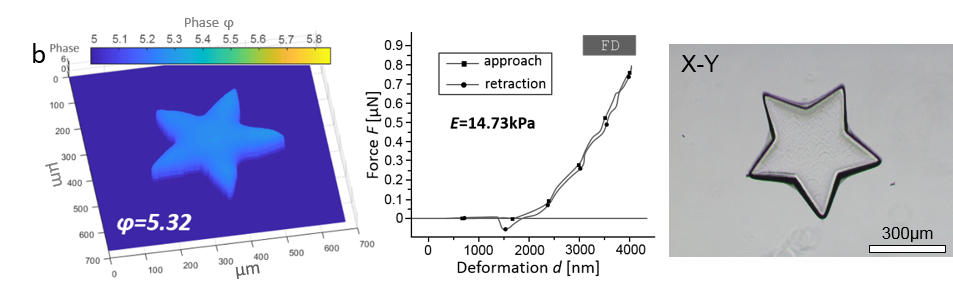


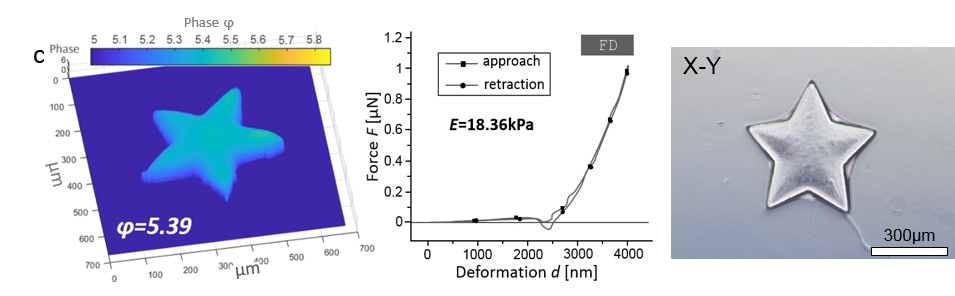


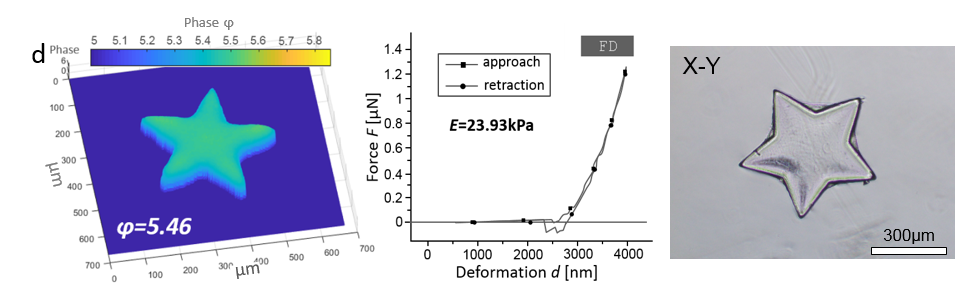


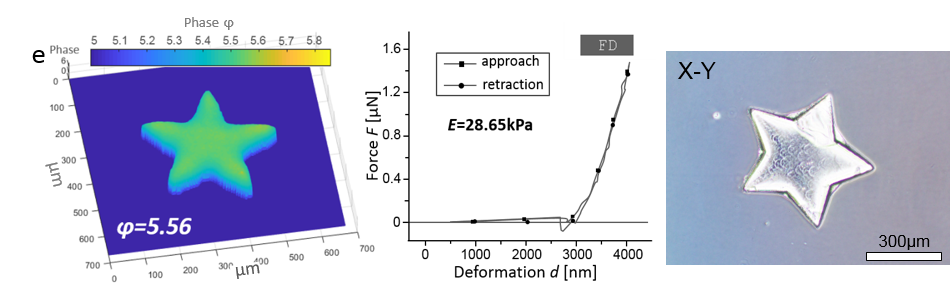


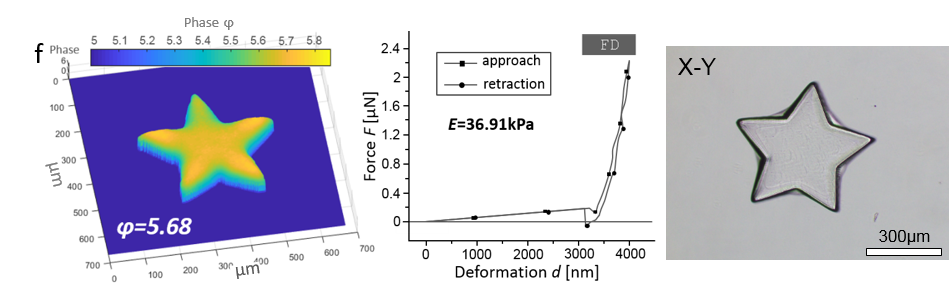


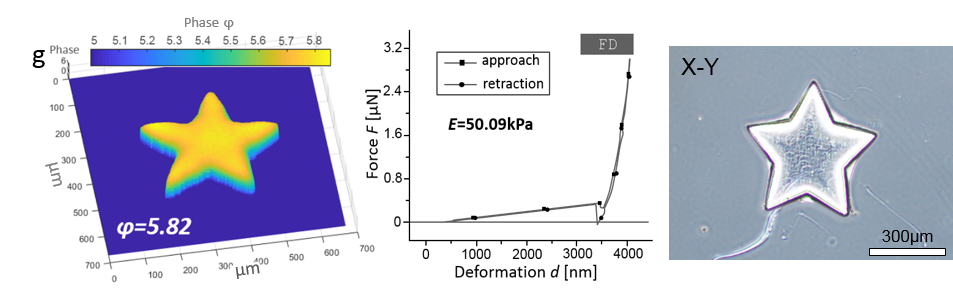


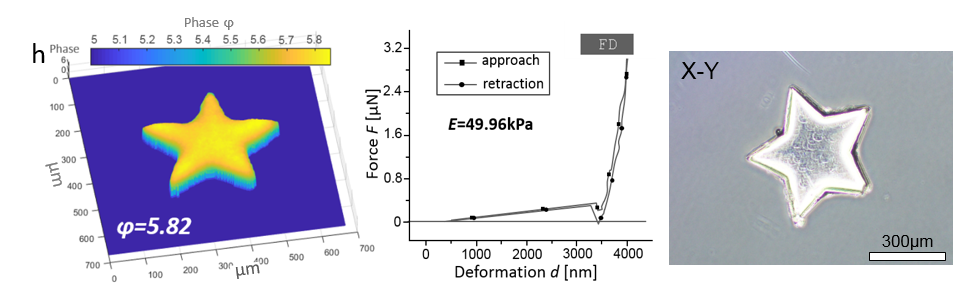


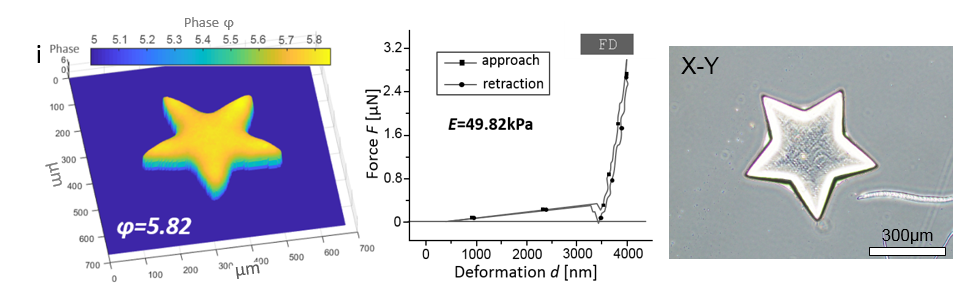


Supplementary Fig. S1. The individual graphs used to fit the mapping relationship between refractive index and Young’s modulus. ϕ: Phase value of structure. *E*: Young’s modulus of structure. The thickness of all these structures are 300 μm.

**
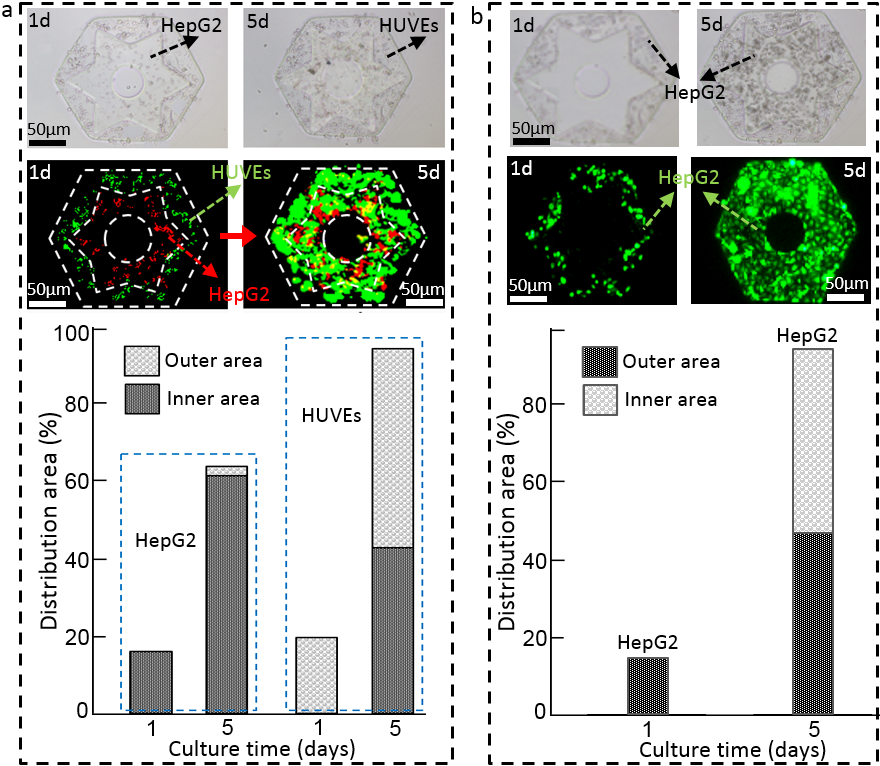
**

Supplementary Fig. S2. a Quantification of HepG2 cells and HUVECs distribution in the inner and outer structures. b HepG2 cells distribution in the micromodules without variation of mechanical stiffness.
